# A proline rich protein from the gingival seal around teeth exhibits antimicrobial properties against *Porphyromonas gingivalis*

**DOI:** 10.1038/s41598-021-81791-7

**Published:** 2021-01-27

**Authors:** Aurélien Fouillen, Charline Mary, Katia Julissa Ponce, Pierre Moffatt, Antonio Nanci

**Affiliations:** 1 Laboratory for the Study of Calcified Tissues and Biomaterials, Faculty of Dental Medicine, Université de Montréal, Montreal, QC Canada; 2 Department of Biochemistry and Molecular Medicine, Faculty of Medicine, Université de Montréal, Montreal, QC Canada; 3 Department of Human Genetics, McGill University, Montreal, QC Canada; 4 Shriners Hospitals for Children - Canada, Montreal, QC Canada

**Keywords:** Proteins, Antimicrobials, Bacteria

## Abstract

The gingival seal around teeth prevents bacteria from destroying the tooth-supporting tissues and disseminating throughout the body. *Porphyromonas gingivalis*, a major periodontopathogen, degrades components of the specialized extracellular matrix that mediates attachment of the gingiva to the tooth. Of these, secretory calcium-binding phosphoprotein proline-glutamine rich 1 (SCPPPQ1) protein has a distinctive resistance to degradation, suggesting that it may offer resistance to bacterial attack. In silico analysis of its amino acid sequence was used to explore its molecular characteristics and to predict its two- and three-dimensional structure. SCPPPQ1 exhibits similarities with both proline-rich and cationic antimicrobial proteins, suggesting a putative antimicrobial potential. A combination of imaging approaches showed that incubation with 20 μM of purified SCPPPQ1 decrease bacterial number (p < 0.01). Fluorescence intensity decreased by 70% following a 2 h incubation of *Porphyromonas gingivalis* with the protein. Electron microscopy analyses revealed that SCPPPQ1 induced bacterial membrane disruption and breaches. While SCPPPQ1 has no effect on mammalian cells, our results suggest that it is bactericidal to *Porphyromonas gingivalis,* and that this protein, normally present in the gingival seal, may be exploited to maintain a healthy seal and prevent systemic dissemination of bacteria.

## Introduction

The moist, warm and nutriment rich environment of the mouth sustains a rich oral microbiome comprising over 700 species^1,2^. However, only some of them have the pathogenic potential to cause periodontal diseases (PD)^3^. Among these, *Porphyromonas gingivalis* (*P. gingivalis*) is a keystone pathogen in PD that is implicated both in its onset and progression^4^. *P. gingivalis* destroys the connective tissues that hold teeth in place, eventually leading to their loss^5^. Also, as PD progress, periodontal bacteria and their products can disseminate throughout the body^6^. More specifically, *P. gingivalis* has been associated with systemic afflictions^7^ such as cardiovascular diseases and respiratory tract infections and recently with Alzheimer disease^8,9^. Unfortunately, there is still no definitive cure against PD and treatment essentially relies on constant intervention to limit bacterial propagation and tissue destruction^10^. There is clearly the need for a better control of *P. gingivalis* both locally and at dissemination sites.

A specialized portion of the gingiva, called junctional epithelium (JE), seals off the tooth supporting tissues from the aggressive environment of the oral cavity^11^. Under healthy conditions, it also prevents the local infiltration of bacteria as well as their dissemination throughout the body^5^. The keratinocytes of this JE produce a unique, adhesive extracellular matrix at the interface between the cells and the tooth surface^12^. While the focus has largely been on the destruction of connective tissue elements, we have recently shown that *P. gingivalis* can also affect the epithelially-derived adhesive matrix^13^. Three of its constituent proteins, Amelotin (AMTN), Odontogenic ameloblast-associated (ODAM) and Laminin-332 (Lam332) are rapidly and completely degraded by *P. gingivalis*^13^. However, secretory calcium-binding phosphoprotein proline-glutamine rich 1 (SCPPPQ1) protein, is conspicuously resistant to degradation by *P. gingivalis*^13^. Therefore, SCPPPQ1 is the only of these 4 proteins implicated in the gingival adhesive “glue”, which is not affected by *P. gingivalis*. This distinctive resistance raises the possibility that, in addition to a structural role, SCPPPQ1 may have another contribution to maintaining a healthy gingival seal.

Throughout evolution, prokaryotic and eukaryotic organisms have developed host defense mechanisms against microbial infections. Among these, naturally derived antimicrobial peptides (AMPs) are particularly important as they may provide a new alternative to antibiotics^14^. Indeed, small AMPs act indirectly by creating intra-bacterial defects, generally by interacting with key proteins or RNA^15^, while the largest AMPs directly attack the bacterial outer membrane leading to important membrane disruptions. Small cationic molecules (< 10 kDa) comprising an important proportion of hydrophobic residues (> 30%) form a category of AMPs^16^. Also, proline-rich AMPs (PrAMPs) are well-known subset of AMPs that disrupt bacterial integrity^17^. In the context of PD, a number of small peptides of variable composition and origin (mammalian, eukaryotic, or purely synthetic), have also been found to be effective at controlling the growth of *P. gingivalis* and/or biofilm formation (reviewed in^18^).

SCPPPQ1 is a small 9 kDa protein encoded by the *scpppq1* gene that localizes to the secretory calcium-binding phosphoprotein (SCPP) gene cluster and is rich in hydrophobic residues such as proline (18%), leucine (16%) and phenylalanine (10%)^19^. Sequence alignment reveals a high homology and conservation of SCPPPQ1 among species^20^. The SCPP gene cluster also encodes for a number of proteins possessing antimicrobial properties^21^, including some well-known AMPs such as Histatin1^14^. This link to the SCPP gene cluster, its proline rich nature, small size, and unique resistance to proteases^13^ suggest that SCPPPQ1 may have antimicrobial capacity. To implicitly eliminate the null hypothesis (H0) that SCPPPQ1 has no antibacterial activity against *P. gingivalis* we have used a two-pronged approach that is, look for structural alterations and test for statistical difference between treatment parameters. Our specific objective was to apply molecular and biophysical techniques to define in silico and in vitro the impact of rat SCPPPQ1 protein on this aggressive bacterium implicated in PD.

## Results

### In silico analysis predicts that SCPPPQ1 may possess antimicrobial properties

**Figure 1:**
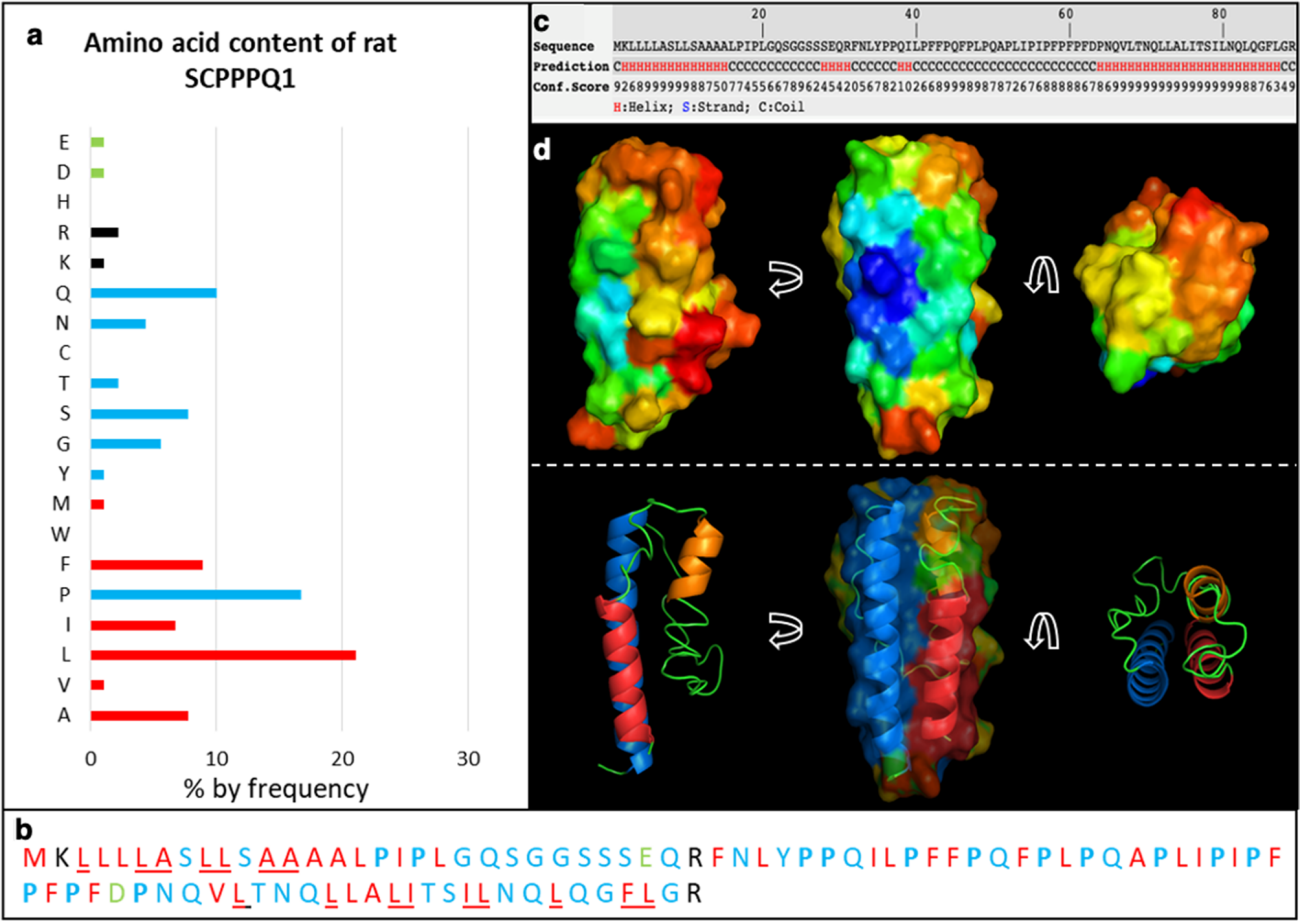
In silico analysis of the rat SCPPPQ1 sequence. (**a**) Analysis of the amino acid content reveals the high hydrophobicity of the protein (40%) and a total net charge of + 1. (red = hydrophobic amino acids; black = positively charged amino acids; green = negatively charged amino acids; blue = other amino acids) (APD3^22^, http://aps.unmc.edu/AP/). (**b**) SCPPPQ1 possesses a sequence rich in proline residues (highlighted in blue and bold). The hydrophobic residues believed to be on a same 3D surface are underlined. (**c**) iTASSER^23^ (https://zhanglab.ccmb.med.umich.edu/I-TASSER/) analysis predicts the position of helices on SCPPPQ1. (**d**) The Quark^24^ (https://zhanglab.ccmb.med.umich.edu/QUARK/) model further predicts that the red ⍺-helix corresponds to signal peptide (residues 2 to 15), the orange ⍺-helix to residues 28 to 32, and the blue ⍺-helix to residues 64 to 87, which is consistent with the iTASSER prediction.

The software APD3^22^ was used to determine the general and antimicrobial properties of the rat SCPPPQ1 sequence. The analysis predicted that the protein possesses antimicrobial properties and may interact with membranes (see output in Fig. S1). The amino acid distribution of the sequence further highlighted the high hydrophobicity of the protein (40%), the presence of 14 prolines and a total net charge of + 1 (Figs. 1a, S1). It also suggested that the protein may form ⍺-helices comprising at least nine residues on the same hydrophobic surface (Fig. S1). iTASSER^23^ and Quark^24^ were used to predict the 3D structure of SCPPPQ1. The results were consistent with the APD3 prediction and assigned the hydrophobic amino acids to stable regions at the surface of the model and the propensity to form ⍺-helices (Fig. 1b–d). Comparisons of alignments with peptides sequences from the APD3 database were further used to determine the percentage of similarities with other known AMPs. The results showed that rat SCPPPQ1 has few similarities with other proteins with the exception of Bactenecin 7 (Bac-7) (sequence alignment shown in Fig. S2), a well-known PrAMP produced by bovine neutrophils^25^.

### Incubation with rat SCPPPQ1 stops bacterial growth

**Figure 2:**
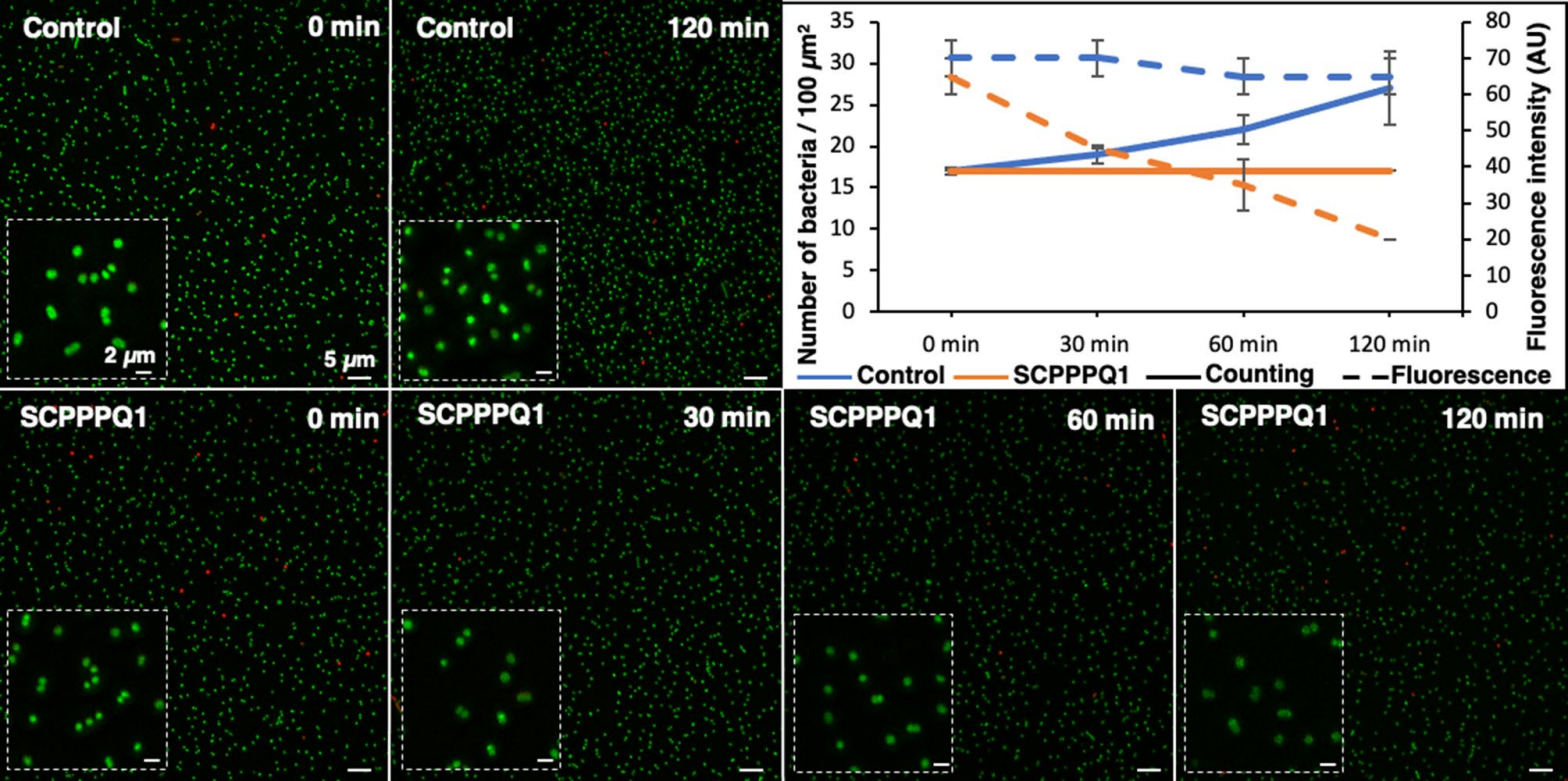
Super-resolution live-and-dead fluorescence imaging of *P*. *gingivalis* incubated with buffer only (control) and SCPPPQ1 (20 µM)*.* Both qualitative visual evaluation (insets) and quantitative evaluation of the fluorescence (also see Fig. S3), show that there are no significant changes in fluorescence intensity throughout the incubation interval for the control, while there is a major drop when cells are incubated with SCPPPQ1. Quantification of the bacteria (upper right panel) shows that between the 0 and 120 min interval the number of bacteria almost doubles whereas with SCPPPQ1 it remains static. In all cases, very few dead cells (red) are evident. Green = Syto9 labelling; Red = Propidium iodide. Data represent the means ± SD (n = 3).

Fluorescence imaging was used to follow the growth of the bacteria in solution over two-hours in the presence or in the absence of purified SCPPPQ1. Super-resolution imaging of *P. gingivalis* stained with Syto9 and propidium iodide revealed no change in fluorescence and a significant increase (p < 0.001) of bacterial numbers (almost doubling) under control conditions (Fig. 2). When incubated with SCPPPQ1, bacteria growth stalled and there was an overall decrease in fluorescence over time (Figs. 2, S3). As a bacterium dies, the Syto9 fluorophore will interact less with nucleic acid resulting in a decrease of the overall fluorescence of cells. Analysis of the gray value (fluorescence intensity) of the super-resolution images indicated a significant (p < 0.01) decrease of the intensity by ~ 30% in the first 30 min, ~ 50% after 1 h and up to 70% after 2 h (Figs. 2, S3). Also, the fact that we did not observed red stained bacteria with propidium iodide indicates that the dying bacteria were not completely permeable. These results suggest that incubation with SCPPPQ1 has a bacteriostatic effect under the conditions tested.

### Rat SCPPPQ1 decreases the adherence capacity of *P. gingivalis*

**Figure 3:**
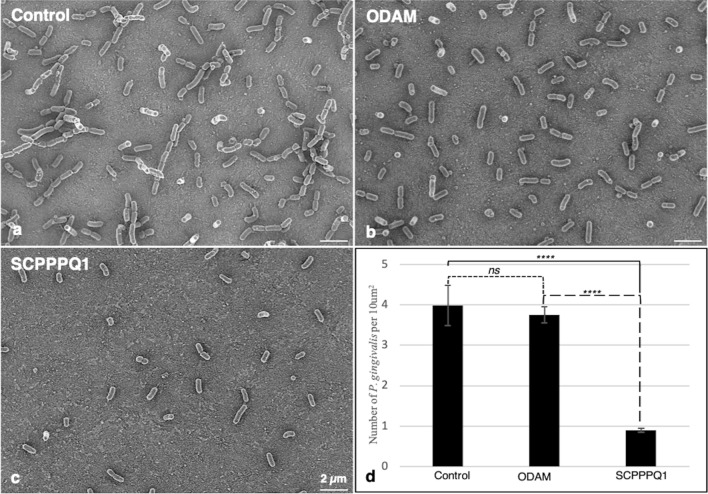
Scanning electron microscopy (SEM) imaging of *P. gingivalis*. Cultures of *P. gingivalis* were incubated in (**a**) the absence (buffer only, control), or presence of (**b**) ODAM (20 µM) or (**c**) SCPPPQ1 (20 µM) for 2 h. Bacteria were then deposited on titanium disks, allowed to adhere for 30 min and processed for SEM imaging. Both qualitative (**a–c**) and quantitative (**d**) analysis reveal an important decrease in the number of *P. gingivalis* when incubated with SCPPPQ1 compared to ODAM or buffer as controls. Representative pictures from one experiment are depicted. *T-test* analysis was used to determine the significance of the quantification in comparison to the control (means ± SD; n = 5). *ns* not significant; ****p < 0.0001.

Scanning electron microscopy (SEM) was used to evaluate the number of bacteria adhering to surface of the titanium sample support following exposure to SCPPPQ1, or its sister protein ODAM and buffer only as controls (Fig. 3a–c). The design of this type of experiment differs slightly from the one presented above in Fig. 2. Rather than continuously monitoring bacterial growth over a 2 h period, it is a snapshot of those bacteria that are still capable of adhering to the titanium surface after exposure. After 2 h of incubation with SCPPPQ1, as compared with ODAM or the buffer only, there was a statistically significant (p < 0.0001) decrease (~ 75%) (Fig. 3d) in the number of bacteria adhering to the SEM support.

### Rat SCPPPQ1 disrupts the cell envelope of *P. gingivalis*

**Figure 4:**
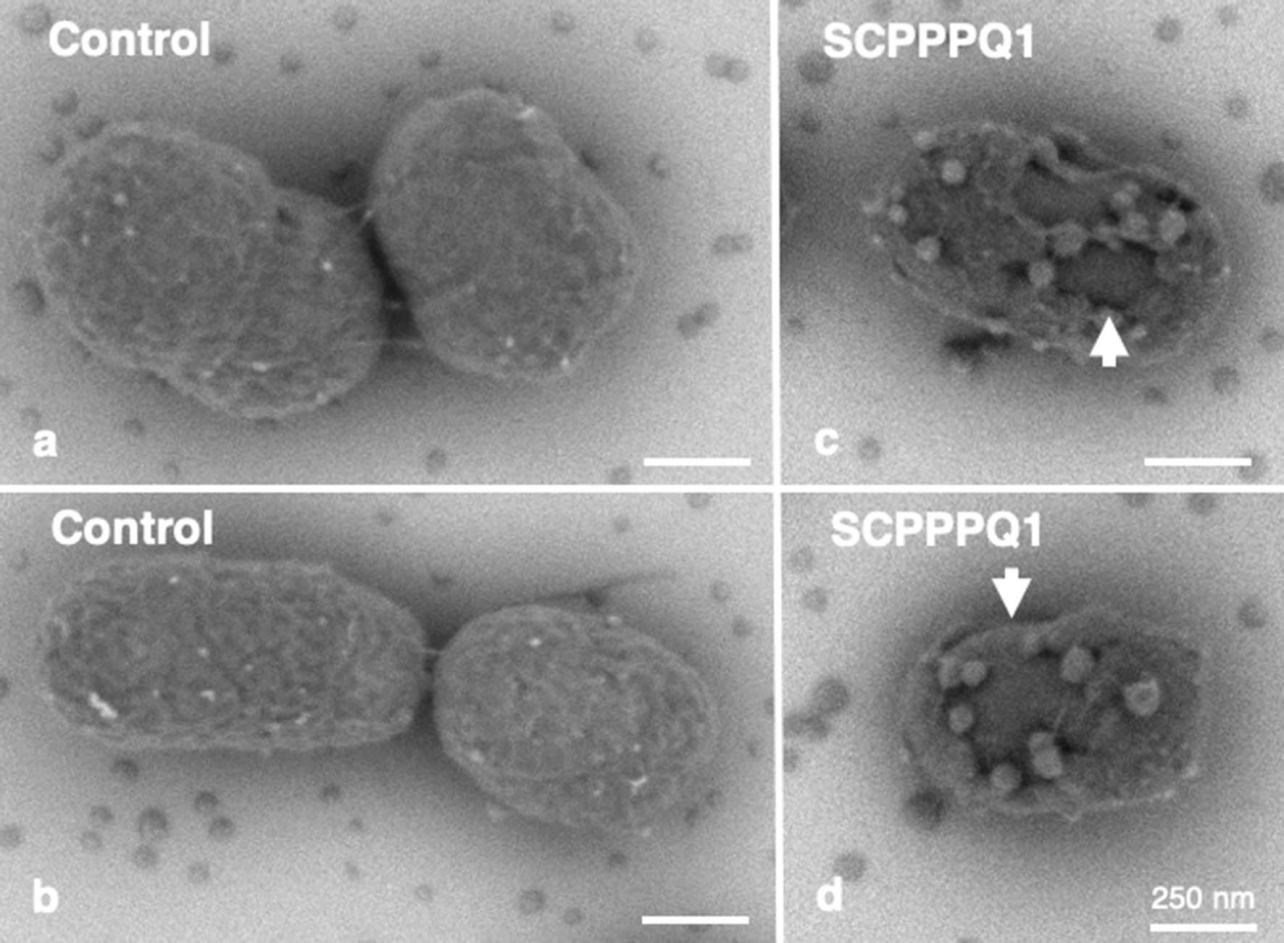
Scanning electron microscopy (SEM) imaging of the bacterial surface. Cultures of *P. gingivalis* were incubated in (**a**,**b**) the absence (buffer only, control) or (**c**,**d**) presence of SCPPPQ1 (20 µM) for 2 h. Bacteria were then deposited on titanium disks, allowed to adhere for 30 min and processed for high-resolution SEM imaging. *P. gingivalis,* incubated with buffer only (**a**,**b**) show intact membranes, while incubation with SCPPPQ1 (**c**,**d**) results in important surface breaches (arrows) and abundant blebbing.

**Figure 5:**
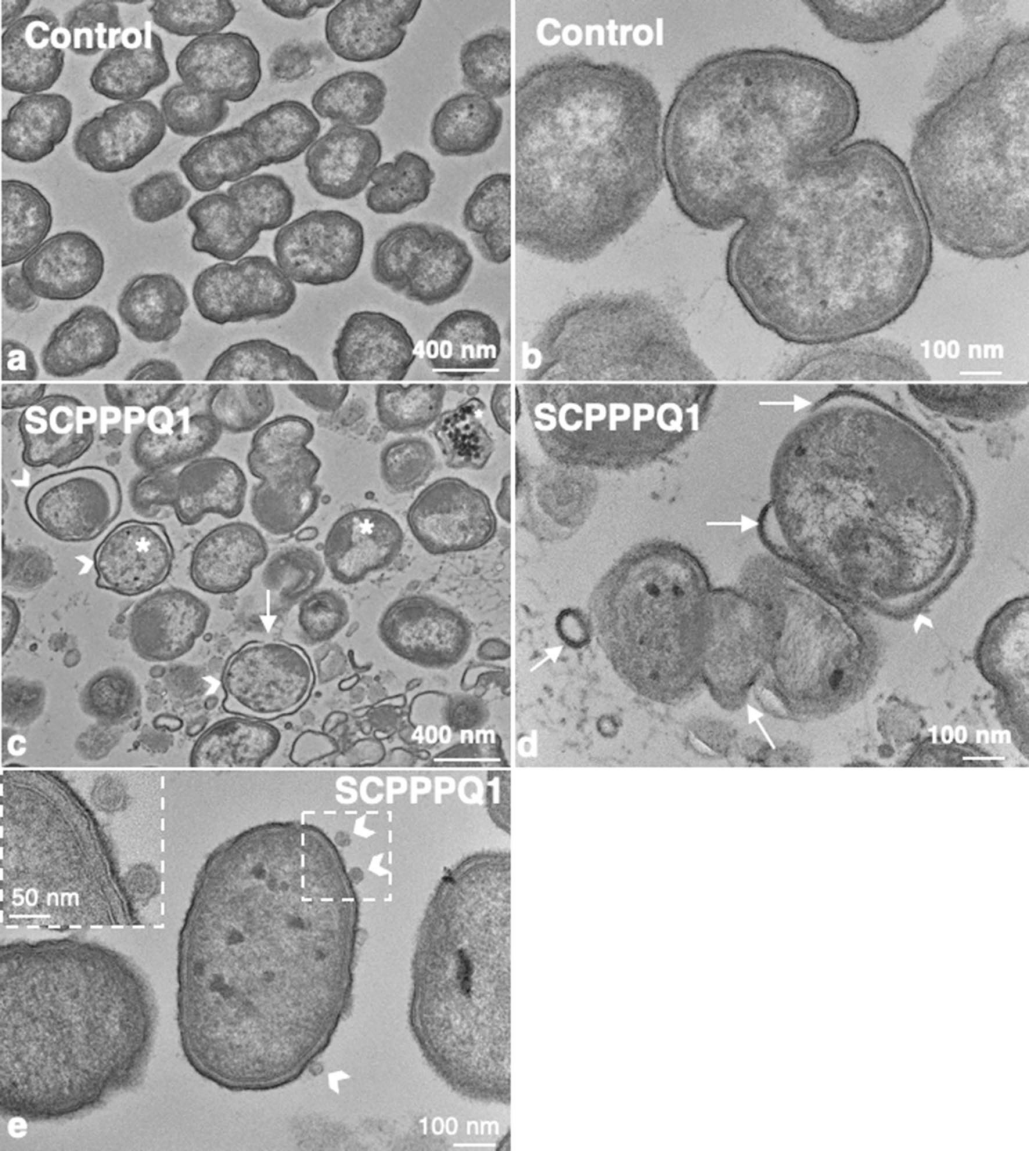
Transmission electron microscopy (TEM) visualization of *P. gingivalis*. Cultures of *P. gingivalis* were incubated in (**a**,**b**) the absence (buffer only, control) or (**c**–**e**) presence of SCPPPQ1 (20 µM) for 2 h. Cultures were fixed and processed for TEM imaging. Comparison *P. gingivalis* incubated with (**a**,**b**) buffer or (**c**–**e**) with SCPPPQ1 reveals that the presence of SCPPPQ1 causes membrane disruptions (arrows), accumulation of dark bodies in the cytoplasm (asterisks) and increases the space between the inner and outer membranes (arrowheads). Debris were also frequently present interspersed between the bacteria in the presence of SCPPPQ1. (**e**) High magnification image showing that bacteria incubated with SCPPPQ1 frequently have outer membrane vesicles on their surface.

Three different electron microscopy approaches were then used to determine the effect of SCPPPQ1 on the bacterial membrane structure and integrity. First, high-resolution SEM imaging suggested that the outer membrane of *P. gingivalis* was affected after incubation with SCPPPQ1 (Fig. 4). Compared to control (Fig. 4a,b), we observed interruptions of the continuity of the cell envelope and abundant blebbing (Fig. 4c,d). We then analyzed thin sections of the bacteria by transmission electron microscopy (TEM). The results clearly confirmed that the outer membrane was affected by SCPPPQ1 (Fig. 5c,d) as compared to the control (Fig. 5a,b). The space between the double-membranes of the bacteria varied (Fig. 5c) and several bacteria showed important outer membrane breaches that are bound to disrupt the integrity of the bacterial cells (Fig. 5d). TEM images revealed that some of the blebbing observed by SEM (Fig. 4) correspond to outer membrane vesicles^26^ (Fig. 5e). There were residues between the cells that may correspond to membrane fragments (Fig. 5c,d). In the presence of SCPPPQ1, some of the bacteria also exhibited accumulation of dark bodies in the cytoplasm (Fig. 5c). Further three-dimensional reconstructions of *P. gingivalis* incubated with SCPPPQ1 obtained by ‘focused ion beam (FIB) and view’ imaging permitted to visualize membrane alterations along the entire bacterial surface (Movies S1, S2). These imaging techniques clearly confirm that SCPPPQ1 can profoundly affect the bacterial membranes.

### Rat SCPPPQ1 is not toxic to mammalian cells

**Figure 6:**
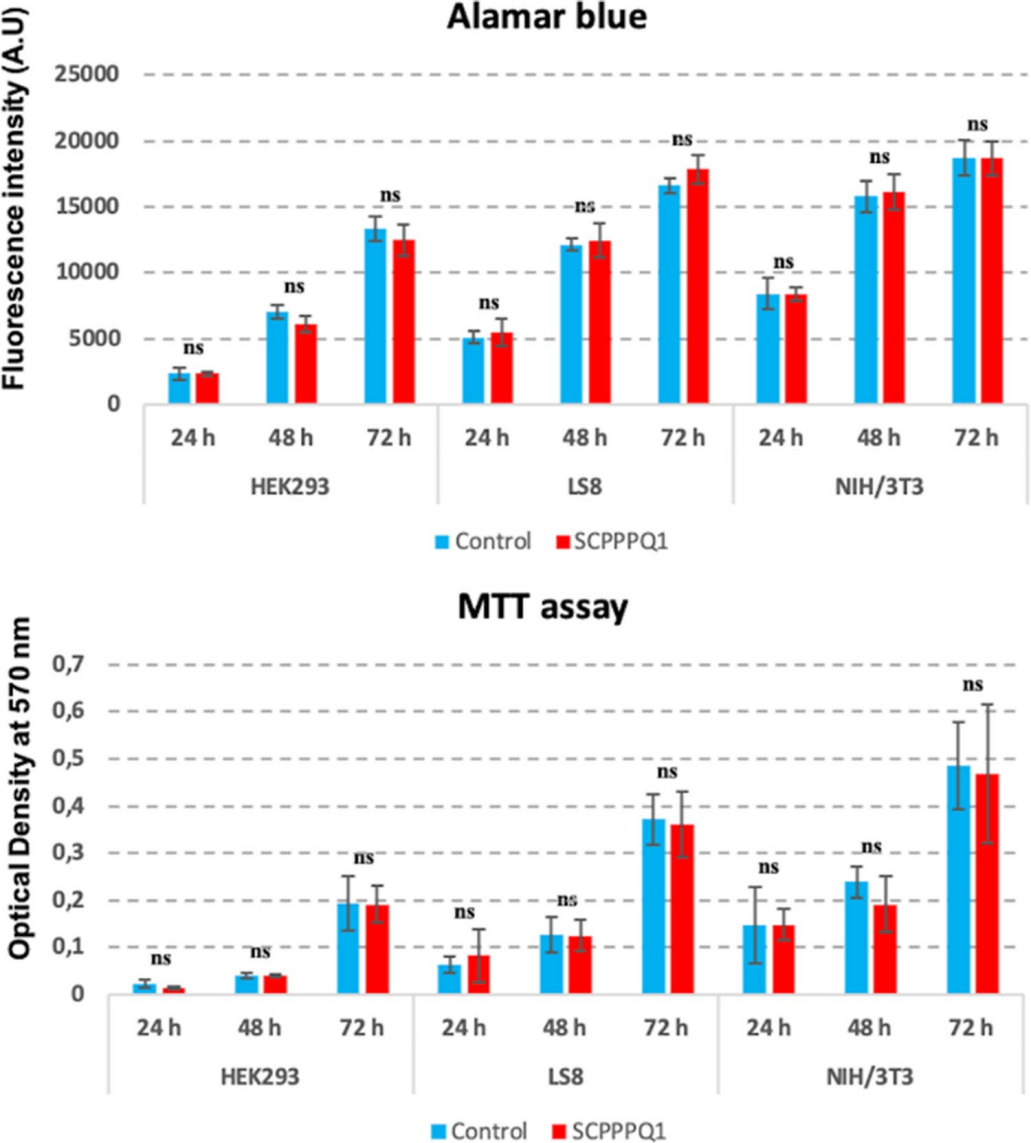
Analysis of the effect of SCPPPQ1 on mammalian cells. HEK293, LS8 and NIH/3T3 cells were incubated with SCPPPQ1 (20 µM) or only the buffer (control) for 72 h. Each 24 h, some cells were processed for both Alamar Blue and MTT assays. The data reveal no significant difference in cell proliferation in the presence or absence of SCPPPQ1. Data represent the means ± SD (n = 3). *A.U.* arbitrary unit, *ns* not significant.

**Figure 7:**
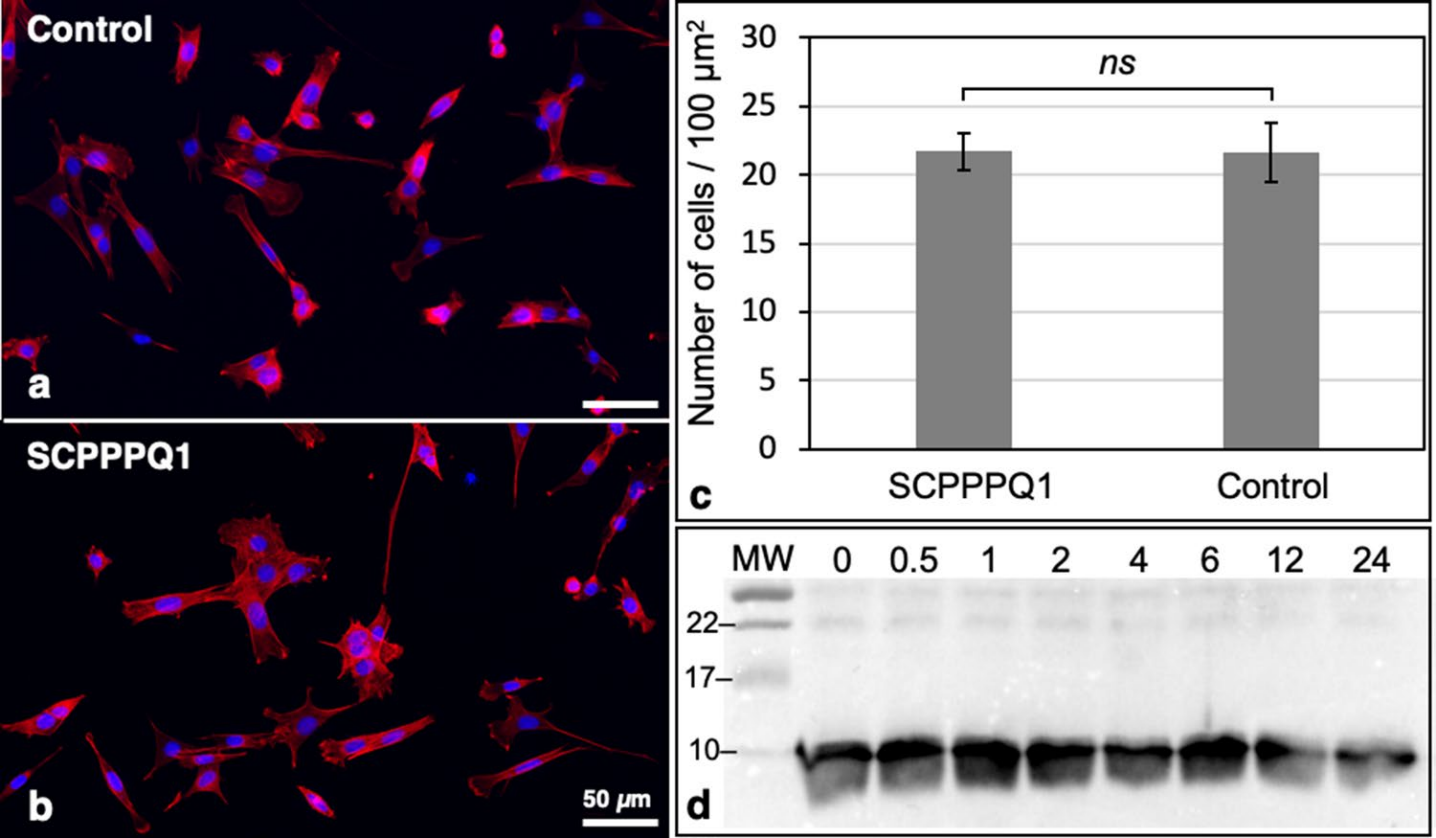
Evaluation of the effect of SCPPPQ1 on LS8 cells. Ameloblast-like LS8 cells were seeded on coverslips, grown overnight, and incubated for 24 h in (**a**) the absence (control) or (**b**) presence of SCPPPQ1 (20 µM) in the media. (**a**,**b**) Cells were fixed and stained with rhodamine phalloidin (red) and Hoechst (blue), and (**c**) images analyzed to assess cell density. Data represent the means ± SD (n = 3). *ns* non-significant. (**d**) The media of cells incubated with SCPPPQ1 was sampled over the 24 h incubation period and analyzed by Western blotting to detect SCPPPQ1. Numbers at top indicate the time in hours and molecular weight (MW) markers (kDa) are labeled at left.

We wanted to make sure that the concentration of the protein used to elicit antibacterial activity does not initiate plasma membrane rupture and has no toxic effects on mammalian cells. There was no significant effect of the presence of SCPPPQ1 on cell proliferation of HEK293, LS8, and NIH/3T3, as compared to the buffer (control) (Fig. 6). We have also sampled the culture media of LS8 cells for up to 24 h and the aliquots were processed for Western blot analysis. The intensity of the band corresponding to SCPPPQ1 did not significantly change throughout the sampling period (Fig. 7d). LS8 cells were also processed for immunofluorescence to highlight the actin cytoskeleton and the nucleus. No readily apparent changes in the cell morphology and number were observed (Fig. 7a–c). These results indicate that cells were not affected by the presence of SCPPPQ1 in the medium during the 24 h culture interval. They also indicate that SCPPPQ1 levels in the media persisted stably throughout the incubation period, suggesting no apparent binding to, or capture by, the mammalian cells tested.

## Discussion

The objective of our work was to better characterize the antibacterial potential of rat SCPPPQ1 protein, a structural matrix molecule produced by the JE that is resistant to bacterial degradation^13^. We have applied fluorescence and electron microscopy to evaluate whether SCPPPQ1 also has antimicrobial activity. The severe structural alterations of the cell membrane of *P. gingivalis* observed when exposed to SCPPPQ1 and the resulting highly significant reduction in population size exclude the null hypothesis and support the alternate hypothesis. This leads us to the conclusion that SCPPPQ1 has a bactericidal effect on this bacterium. Because *P. gingivalis* is a keystone periodontopathogen during the onset and progression of PD^4^, and because it has been linked to a number of systemic afflictions^7^, SCPPPQ1 may also be part of the complex strategies devised by the body to control and prevent bacterial dissemination^27^.

SCPPPQ1 belongs to the SCPP genes cluster^19^ that also encodes for some well-known salivary AMPs^14,28^ and some milk-AMPs^21^. SCPPPQ1 can be classified as a cationic AMP^29^ and shares similarities with some extensively studied PrAMPs^30^, noteworthy with Bac-7, an AMP which disrupts Gram-negative bacteria^31^. Proteins from both of these classes adopt conformations that favor interaction with the negatively-charged bacterial membranes^32^ and cause membrane breaches^33^. As we show here, both SEM and TEM support the concept that the primary mechanism of action of SCPPPQ1 is a direct alteration of the bacterial outer membrane, a capacity consistent with the mode of action of both PrAMPs and cationic AMPs. Its resistance to *P. gingivalis* however highlights the unique nature of SCPPPQ1 and places it in a distinctive subset of AMPs. Like for smaller AMPs, in addition to the destruction of the outer bacterial membrane, it could also be envisaged that the smaller active portion of SCPPPQ1 could directly penetrate the cells to enhance the efficacy of the anti-bacterial activity^34^.

While keratinocytes produce AMPs to prevent infections of skin wounds^35^, only cells of the JE produce SCPPPQ1^20^. The JE deposits a unique extracellular matrix at the interface with the tooth^36^. The proteins constituting this matrix interact to form a supramolecular network in which they are differentially distributed, with SCPPPQ1 tending towards the tooth surface^12^. As PD set in, bacteria such as *P. gingivalis*, could cleave/degrade AMTN, ODAM and Lam332^13^. This is supported by clinical research showing that digested portions of ODAM and Lam332 are released into the gingival space above the JE in patients with advanced periodontitis^37,38^. Such a degradation is bound to directly expose SCPPPQ1 to the bacteria, a situation comparable to AMPs that accumulate at infected sites, such as defensins in the skin^27^. However, SCPPPQ1 is not continuously produced by the cells of the JE under healthy conditions^20^. Therefore, its production may not be sufficient during the progressive phase of PD, during which the ever-increasing number of bacteria of the dental biofilms, and not only *P. gingivalis*, could overwhelm the available SCPPPQ1. As it has been shown for the sister protein AMTN^39^ that has no antibacterial capacity^13^, inflammatory factors produced during disease could stimulate production of SCPPPQ1 and thereby increase its available concentration. Under this circumstance, adding the protein or derived peptides at therapeutic doses may ultimately be the best strategy to both prevent and counteract PD progression. Despite the fact that *P. gingivalis* is a keystone bacterium in PD, it will be necessary to further characterize the effect of SCPPPQ1 on a larger spectrum of bacteria, and even on fungi such as *Candida albicans*, which can all have a role in PD. Indeed, the dental plaque is a polymicrobial environment comprising several kinds of bacteria more or less aggressive. Our study is thus a first step to address the broader antimicrobial potential of SCPPPQ1.

Conventional antibiotic treatments have shown the capacity to temporarily slow down PD progression, but their beneficial effects are not sustained over time^40^. There are other molecules such as chloromethane compounds that are very efficient against *P. gingivalis* in vitro^41^, but these damage mammalian cells^41^ and are therefore not optimal for in vivo therapy. Interestingly, SCPPPQ1 does not affect mammalian cells (Figs. 6, 7). The lack of cell toxicity is actually expected given the fact that SCPPPQ1 is naturally-produced and present in the JE epithelium of all individuals. Even if further studies are needed, the inherent exposure of oral bacteria to SCPPPQ1 is likely to limit any bacterial resistance resulting from its therapeutic use. It must be mentioned, however, that a number of short peptides with sequences distinct from SCPPPQ1 have been found to be effective at killing *P. gingivalis* and/or preventing biofilm formation^18^. For instance, Pac-525 (KWRRWVRWI) is a tryptophane-rich peptide-derivative of the bovine neutrophil granule indolicidin^42^. It was shown to inhibit the growth of *P. gingivalis* with a minimal effective concentration of 62.5 μg/ml, and to prevent adhesion to titanium surfaces at 500 μg/ml. The P-113 peptide (AKRHHGYKRKFH), derived from the saliva protein Histatin 5, showed bactericidal activity on *P. gingivalis* at 320 μg/ml in the planktonic state, or at 1280 μg/ml when used on biofilms^34^. Lastly, two other peptides derived from the streptococcal proteins SspB (LEAAPKKVQDLLKKANITVKGAFQLFS)^43^ and ArcA (NIFKKNVGFKK)^44^ were found effective at 4 μg/ml and 32 μg/ml, respectively, to kill and reduce virulence of *P. gingivalis*.

While periodontopathogens are typically associated with oral manifestations such as PD, they are now being increasingly implicated in major systemic complications^6^. Conventional antibiotic therapies have not proven efficient and there is still no treatment for the control of both periodontal and associated systemic afflictions. In the past 10 years, several studies have linked PD, and more specifically *P. gingivalis,* with Alzheimer disease^8,9,45^. Dominy et al.^8^ suggested that “gingipains were neurotoxic in vivo and in vitro, exerting detrimental effects on tau” and thus exacerbating amyloid plaque formation, a key factor of Alzheimer disease development. While they reported exciting outcomes concerning the inhibition of gingipains^46^, a group of cysteine proteinases, controlling them in the brain could be a challenging endeavor and may require continuous treatments. Furthermore, the origin of gingipains in human brain needs further clarification. They could originate locally in the brain from invading bacteria or they could be translocated via membrane vesicles from the periodontal space. Irrespectively of the origin of the vesicles, it would seem that, in both cases, controlling the source of the problem, that is the bacteria itself, might ultimately represent a desirable prospect. While Alzheimer is a complex disease, if the link to *P. gingivalis* bears through, a better control of PD is certainly expected to translate into a concurrent impact at the brain level.

We have shown that SCPPPQ1 possesses antimicrobial activity, yet there are still several issues that need to be explored in future studies. First, it remains to be determined if the whole SCPPPQ1 protein is necessary to exhibit its activity. Alternatively, the activity could reside within a short peptide segment of the protein, whether it be the central proline-rich domain, or the alpha-helix located in the C-terminus. The identification of the active portion will also perhaps allow to further explain the molecular mechanism by which it can disrupt the bacterial membrane, and be compared to established scenarios put forward for other AMPs^47–49^. This will further guide the optimization of more effective peptides for controlling bacterial pathogens, whereby smaller active peptides could be more potent. While the concentration used in the current study (20 μM; 160 μg/ml) has proven very efficient to repress bacterial growth, a direct experimental comparison of the antimicrobial effects between SCPPPQ1 and known AMPs will eventually need to be conducted to determine whether it is superior or not. It is difficult, however, to relate the concentration used in vitro with that produced locally by cells of the JE in vivo, and whether SCPPPQ1 could resist attacks by *P. gingivalis* in the complex physiological environment of the oral cavity. It can also be questioned if SCPPPQ1 would have the same antibacterial potential when tested on mixtures of pathogens present in dental plaques. The development of a mouse model lacking the *scpppq1* gene (under way in our laboratory) will be key to confirm its antimicrobial role in vivo. Likewise, the model will also provide an opportunity to validate if exogenously administered SCPPPQ1 protein or peptides may be able to slow down breakdown of the gingival seal, the onset of PD, and associated systemic complications.

In conclusion, our results show for the first time that a small, oral keratinocyte-derived protein, SCPPPQ1, has antibacterial activity against *P. gingivalis*. Because of its endogenous origin and its non-toxicity on mammalian cells, it represents an excellent candidate for limiting the progression of PD, but also to deal with systemic complications related to *P. gingivalis*. Small AMPs disseminate better, are generally more efficient and stable, and are less prone to degradation^50^, all major assets in the fight against bacteria. While SCPPPQ1 is a small protein, identification of its active portions will allow optimization of its effectiveness and of its delivery, particularly across the blood brain barrier.

## Methods

### In silico analysis

The AMP database APD3^22^ (http://aps.unmc.edu/AP/, University of Nebraska, Omaha, NE, USA) was used to determine the antimicrobial potential of SCPPPQ1. The rat SCPPPQ1 sequence was analyzed to evaluate the peptide length, its net charge, its amino acid composition, the secondary structure and its sequence similarity with other AMPs. Quark^24^ and iTasser^23^ (https://zhanglab.ccmb.med.umich.edu/, University of Michigan, Ann Arbor, MI, USA) were used individually to predict the tridimensional (3D) conformation. MacPyMOL (The PyMol Molecular Graphics System, Version 2.0 Schrödinger, LLC, New York, NY, USA) was finally used to analyze the model and to highlight the position of the hydrophobic residues.

### Cloning procedures

The sister protein ODAM^51^, also belonging to the SCPP cluster, was used as control. Truncated versions of *SCPPPQ1* and *ODAM* genes lacking regions encoding the predicted N-terminal signal sequence were PCR-amplified from rat cDNA sequences using primers as previously described^12^. PCR products were cloned into the vector pHT for purification studies^35^. The recombinant pHT plasmids allow to produce recombinant proteins with an in-frame N-terminal hexahistidyl-tag (His-tag) and TEV protease cleavage site. *Escherichia coli* strain XL-1 Blue were used as hosts for cloning^52^.

### Protein overexpression and purification

The protein overexpression protocol is similar to the ones used in Fouillen et al.^12,13^. Briefly, BL21(DE3)-star cells containing either pHT-*SCPPPQ1* or pHT-*hODAM*^12^ were grown at 37 °C and 250 rpm to an optical density at 660 nm (OD_660_) of around 0.6, and protein expression was induced with 0.1 mM isopropyl-ß-d-thiogalactoside (Thermo Fisher Scientific, Waltham, MA, USA) overnight at 30 °C and 250 rpm. For SCPPPQ1, bacterial cells were harvested, suspended in equilibration buffer (50 mM Na_2_HPO_4_ (Thermo Fisher Scientific, Waltham, MA, USA), 150 mM NaCl (Thermo Fisher Scientific, Waltham, MA, USA), 10 mM imidazole (Sigma-Aldrich, Saint-Louis, MO, USA), 8 M urea (Thermo Fisher Scientific, Waltham, MA, USA), pH 7) at 4 °C, and sonicated 6 × 15 s with 15 s ice incubations in between. Lysates were centrifuged at 13,400 × *g* and the 6His-tagged protein in the supernatant was bound on nickel-nitriloacetic acid (Ni–NTA)-agarose affinity resin (Qiagen, Hilden, Germany) at room temperature. After washing the resin with 10 volumes of binding buffer (50 mM Na_2_HPO_4_ (Thermo Fisher Scientific, Waltham, MA, USA), 300 mM NaCl (Thermo Fisher Scientific, Waltham, MA, USA), 20 mM imidazole (Sigma-Aldrich, Saint-Louis, MO, USA), 8 M Urea (Thermo Fisher Scientific, Waltham, MA, USA), pH 7), proteins were eluted with elution buffer (50 mM Na_2_HPO_4_ (Thermo Fisher Scientific, Waltham, MA, USA), 300 mM NaCl (Thermo Fisher Scientific, Waltham, MA, USA), 300 mM imidazole (Sigma-Aldrich, Saint-Louis, MO, USA), 8 M Urea (Thermo Fisher Scientific, Waltham, MA, USA), pH 7). Concentration of the collected fractions were assessed using a BioDrop (Montreal Biotech, Montréal, Canada) and fractions were then analyzed by sodium dodecyl sulfate–polyacrylamide gel electrophoresis (SDS–PAGE) and Coomassie blue staining. Proteins were finally dialyzed into 50 mM Na_2_HPO_4_ (Thermo Fisher Scientific, Waltham, MA, USA)_,_ 8 M Urea (Thermo Fisher Scientific, Waltham, MA, USA) (pH 7.2) and stored at 4 °C. ODAM was purified in the same conditions as SCPPPQ1 but in a buffer without Urea. Western blot gels were acquired using a Bio-Rad ChemiDoc imager and the software Image Lab version 6.1 (Bio-Rad, Hercules, CA, USA). Exposure time for the acquisition were between 1 and 2 s.

### Bacterial culture and assays

*Porphyromonas gingivalis* ATCC 33277 (ATCC, Manassas, VA, USA) were grown anaerobically for at least 24 h (80% N_2_, 10% CO_2_, 10% H_2_) at 37 °C in Todd-Hewitt broth (THB) (Thermo Fisher Scientific, Waltham, MA, USA) supplemented with 0.001% hemin (Thermo Fisher Scientific, Waltham, MA, USA) and 0.0001% vitamin K (Thermo Fisher Scientific, Waltham, MA, USA). 20 µM of each of the purified proteins (SCPPPQ1 and ODAM) were exposed in a test tube to 800 µl of a suspension of bacteria at a OD_660_ of 1 (CFU = 10^9^ cells/ml^13^) for 2 h at 37 °C. This concentration of *P. gingivalis* was previously used to experimentally induce periodontitis in mice^38^. The protein concentration of 20 μM (160 μg/ml) was selected based on our own prior work showing resistance of SCPPPQ1 to degradation by *P. gingivalis*^13^. Also, it represented a concentration within the range used in other studies looking at the effects of antimicrobial effects of peptides^17,18,34,42,43^. The mixture was then sampled for fluorescence microscopy, SEM and TEM characterization. As a negative control, buffer without the proteins was incubated with *P. gingivalis* under the same conditions.

### Scanning electron microscopy

The SEM protocol is similar to the one described in Fouillen et al.^13^. Following 2 h incubations in a 1.5 ml tube, bacterial samples were applied to polished grade II titanium supports for 30 min. The bacteria that adhered to the support were fixed for 1 h at 4 °C with 4% paraformaldehyde (Thermo Fisher Scientific, Waltham, MA, USA) and 0.1% glutaraldehyde (Electron Microscopy Sciences, Hatfield, PA, USA) in 0.1 M phosphate buffer (PB, pH 7.3), and subsequently rinsed three times with PB. The samples were then post-fixed for 1 h with 1% osmium tetroxide (Electron Microscopy Sciences, Hatfield, PA, USA), dehydrated through an ethanol series (30%, 50%, 70%, 90%, 95% and two times 100%) followed by drying using a Critical Point Drier CPD300 (Leica Biosystems, Concord, ON, Canada). A high-resolution field emission (FE)-SEM Regulus 8230 (Hitachi High-Tech Corporation, Tokyo, Japan), operated at 0.8 kV, was used for imaging the uncoated samples. For each condition, counting of at least 50 representative images, from four independent experiments were analyzed using ImageJ version 1.50i (NIH, Bethesda, MD, USA).

### Immunofluorescence studies

Some bacteria were stained using the Live/Dead BacLight kit (Thermo Fisher Scientific, Waltham, MA, USA) before incubation with SCPPPQ1. Briefly, 1.5 µl of Syto9 and 1.5 µl of propidium iodide were added to 800 µl of bacteria at an OD_660_ of 1. Two hundred µl of protein were added to the mixture in order to obtain a final concentration of protein of 20 µM. As a control, only the buffer was added to the bacterial suspension. During 2 h of incubation, the suspensions were sampled at 15, 30, 60 and 120 min. After each sampling, the mixture was fixed using 1% glutaraldehyde (Electron Microscopy Sciences, Hatfield, PA, USA) to obtain a final concentration of 2.5%. Ten µl of each suspension were finally examined with an Elyra PS1 microscope (Carl Zeiss Microscopy, Oberkochen, Germany) equipped with 63× oil objective (numerical aperture 1.4) and an EMCCD iXon3 DU-885K camera (Andor, Connecticut, USA). Z-stack volumes were acquired using the Structural Illumination Method (SIM) and reconstructed using the Zen Black software version 2.1 (Carl Zeiss Microscopy, Oberkochen, Germany). For each condition, counting and intensity analysis of at least 50 bacteria for each of 20 representative images (5 samples per conditions) from three independent experiments were analyzed using ImageJ version 1.50i (NIH, Bethesda, MD, USA).

### Embedding procedures

Some bacteria were fixed as above, post-fixed with potassium ferrocyanide-reduced osmium tetroxide (Electron Microscopy Sciences, Hatfield, PA, USA) and then processed for embedding in Epon resin (Electron Microscopy Sciences, Hatfield, PA, USA). Ultrathin sections of 80–100 nm were cut with a diamond knife on an Ultracut EM UC6 ultramicrotome (Leica Biosystems, Concord, ON, Canada) and transferred onto Formvar-coated 200-mesh nickel grids for TEM imaging. The grid-mounted sections were examined in a Tecnai 12 TEM (FEI (now Thermo Fisher Scientific), Eindhoven, Netherlands) operating at 80 kV. Some embedded samples were used tomographic imaging using a Crossbeam 550 Focused Ion Beam (FIB)-SEM (Carl Zeiss Microscopy, Oberkochen, Germany). Samples were serially milled at 4 nm thickness using a probe current of 1.5 nA/30 kV. Exposed surfaces were observed at 1.5 kV using Secondary Electron and Energy Selective Backscatter detectors. The Dragonfly software 2020.1 (Object Research Systems, Montréal, Canada) was used for further alignment and 3D reconstruction of the FIB-SEM image stacks.

### Cell analysis

Although SCPPPQ1 is naturally-produced and present in the JE epithelium of all individuals, we wanted to make sure that the protein has no effect against eukaryotic cells. To target the two cell lineages found in the oral cavity (epithelial, fibroblastic) that can be in direct contact with SCPPPQ1, we have used as representative cells LS8 ameloblast-derived epithelial cells^53^ (ATCC, Manassas, VA, USA), and NIH/3T3 fibroblast cells^54^ (ATCC, Manassas, VA, USA). More conventional human embryonic kidney (HEK293) epithelial cells^55^ (ATCC, Manassas, VA, USA) were also used. Cells were cultured in DMEM supplemented with 10% fetal bovine serum (FBS, Thermo Fisher Scientific, Waltham, MA, USA) at 37 °C in a 5% CO_2_ atmosphere. 5000 cells were placed in each well of 96-well plates and grown overnight. The culture medium was then exchanged with fresh one containing either SCPPPQ1 at a final concentration of 20 µM or the equivalent volume of buffer as control. Cells were then grown up to 72 h. After 24 h, 48 h and 72 h of incubation, some cells were processed for an Alamar Blue assay (Thermo Fisher Scientific, Waltham, MA, USA) and others for a MTT assay (3-(4,5-Dimethylthiazol-2-yl)-2,5-Diphenyltetrazolium Bromide) (Thermo Fisher Scientific, Waltham, MA, USA) following company instructions for respective assays. Briefly, for Alamar Blue, the cell media was replaced by 90 µl of DMEM without phenol red (Thermo Fisher Scientific, Waltham, MA, USA) and 10 µl of the Alamar Blue HS solution and placed for 4 h at 37 °C in a 5% CO_2_ atmosphere. Fluorescence of the 96-wells plates were then read at a wavelength of 570 nm. For MTT, the cell media were replaced by 100 µl of DMEM without phenol red and 10 µl of a PBS solution containing 12 mM of MTT. The 96-well plates were then placed for 4 h at 37 °C in a 5% CO_2_ atmosphere, before adding 100 µl of a SDS-HCl solution to each well and to incubate the microplate for an additional 4 h at 37 °C in a 5% CO_2_ atmosphere. Finally, the samples were mixed a last time before reading their absorbance at 570 nm with a 2104 EnVision Multilabel Plate Reader (PerkinElmer, Waltham, MA, USA).

### LS8 cells immunofluorescence staining and western blotting

Ameloblast-like cells LS8 were cultured in DMEM supplemented with 10% FBS at 37 °C in a 5% CO_2_ atmosphere. 250,000 cells were placed on coverslips and grown overnight. SCPPPQ1 was added to the cell media at a final concentration of 20 µM. The same volume of buffer was added as control to some coverslips. After 24 h of incubation, the cells were stained with rhodamine phalloidin and Hoechst (1:500; 1:2000 respectively) (Thermo Fisher Scientific, Waltham, MA, USA) to highlight actin and nuclei. Samples were acquired on an Axio Imager (Carl Zeiss Microscopy, Oberkochen, Germany) using the Zen software version 2.1 (Carl Zeiss Microscopy, Oberkochen, Germany) and the images were analyzed using ImageJ version 1.50i (NIH, Bethesda, MD, USA). Cell media of LS8 cells were collected multiple times during a 24 h culture period and then aliquots were analyzed using SDS-PAGE followed by Western Blot using anti-SCPPPQ1 antibodies (1:1000). Western blot gels were acquired at an exposure times between 1 and 2 s using a ChemiDoc imager (Bio-Rad, Hercules, CA, USA).

### Statistical analysis

For fluorescence, SEM, and cell analysis, values and standard deviations were calculated from at least three independent experiments, and the *p* values were obtained by *t* test analysis of each condition from data in Excel (Microsoft Windows, Albuquerque, NM, USA). Statistical significance was defined as ns, p > 0.05; *p < 0.05; **p < 0.01; ***p < 0.001; and ****p < 0.0001.

## Supplementary Information

Supplementary Information 1.

Supplementary Video 1.

Supplementary Video 2.

## Data Availability

The datasets and/or analyses generated during the current study are available from the corresponding author, Dr. Antonio Nanci, upon reasonable request.
