# Supplementary material for: A proline rich protein from the gingival seal around teeth exhibits antimicrobial properties against Porphyromonas gingivalis
Source: Sci Rep. 2021 Jan 27;11:2353. doi: 10.1038/s41598-021-81791-7 (PMC7840901; doi:10.1038/s41598-021-81791-7)
Supplement: Supplementary file 1 — Supplementary Information 1. [file 41598_2021_81791_MOESM1_ESM.pdf]

# A proline rich protein from the gingival seal around teeth exhibits antimicrobial properties against *Porphyromonas gingivalis*

Aurélien Fouillen[1,2], Charline Mary[1,2], Katia Julissa Ponce[2], Pierre Moffatt[3,4] and Antonio Nanci[1,2*].

[1] Laboratory for the Study of Calcified Tissues and Biomaterials, Faculty of Dental Medicine and [2] Department of Biochemistry and Molecular Medicine, Faculty of Medicine, Université de Montréal, Montréal, Québec, Canada. [3]Department of Human Genetics, McGill University and [4]Shriners Hospitals for Children - Canada, Montréal, Québec, Canada.

* Corresponding author: Antonio Nanci

**Email:** antonio.nanci@umontreal.ca

**Keywords**
SCPPPQ1; Antimicrobial; Porphyromonas gingivalis; Junctional epithelium; Periodontal disease

You input sequence is: LPIPLGQSGGSSSEQRFNLYPPQILPFFPQFPLPQAPLIPIPFPFPFDPNQVLTNQLLALITSILNQLQGFLGR

L-P-I-P-L-G-Q-S-G-G-S-S-S-E-Q-R-F-N-L-Y-P-P-Q-I-L-P-F-F-P-Q-F-P-L-P-Q-A-P-L-I-P-I-P-F-P-F-P-F-D-P-N-Q-V-L-T-N-Q-L-L-A-L-I-T-S-I-L-N-Q-L-Q-G-F-L-G-R-

L P I P L G Q S G G S S S E Q R F N L Y P P Q I L P F F P Q F P L P Q A P L I P I P F P F P F D P N Q V L T N Q L L A L I T S I L N Q L Q G F L G R

L P I P L G Q S G G S S S E Q R F N L Y P P Q I L P F F P Q F P L P Q A P L I P I P F P F P F D P N Q V L T N Q L L A L I T S I L N Q L Q G F L G R

**Hydrophobic residues are in red, hydrophobic residues on the same surface are underlined.**

**Total hydrophobic residues on the same surface is 9**

Your peptide may form alpha helices and it may have at least 9 residues on the same hydrophobic surface.
Your peptide may interact with membranes and has a chance to be an antimicrobial peptide.

**Figure S1**. Results from the analysis of the rat SCPPPQ1 sequence in the APD3 software reveal that the protein may form alpha helices, has 9 residues on the same hydrophobic surface, and has an AMP potential.

**Sequence alignment between rat SCPPPQ1 and Bac-7**

| | | | | | | | | | | | | | | | | | | | | | | | | | | | | | | |
|---|---|---|---|---|---|---|---|---|---|---|---|---|---|---|---|---|---|---|---|---|---|---|---|---|---|---|---|---|---|---|
| *Bac-7* | R | P | R | P | L | P | F | P | R | P | G | + | + | + | + | P | R | + | + | + | + | P | I | P | R | P | L | P | + | F |
| *rat SCPPPQ1* | L | P | I | P | L | + | G | Q | S | G | G | S | S | S | E | Q | R | F | N | L | Y | P | + | P | Q | I | L | P | F | F |
| *Bac-7* | P | + | R | P | G | P | + | R | P | + | I | P | R | P | L | P | F | P | + | R | P | + | + | + | + | + | + | + | + | + |
| *rat SCPPPQ1* | P | Q | F | P | L | P | Q | A | P | L | I | P | I | P | F | P | F | P | F | D | P | N | Q | V | L | T | N | Q | L | L |
| *Bac-7* | + | G | P | R | P | I | + | + | + | + | P | R | P | L | + | | | | | | | | | | | | | | | |
| *rat SCPPPQ1* | A | L | I | T | S | I | L | N | Q | L | Q | G | F | L | G | | | | | | | | | | | | | | | |

**Figure S2**. Alignment between rat SCPPPQ1 and Bac-7 sequence using the APD3 database. The sequence analysis reveals a significant degree of similarity (~26%). Identical residues are indicated in red. Similar amino acids are indicated with a green +.

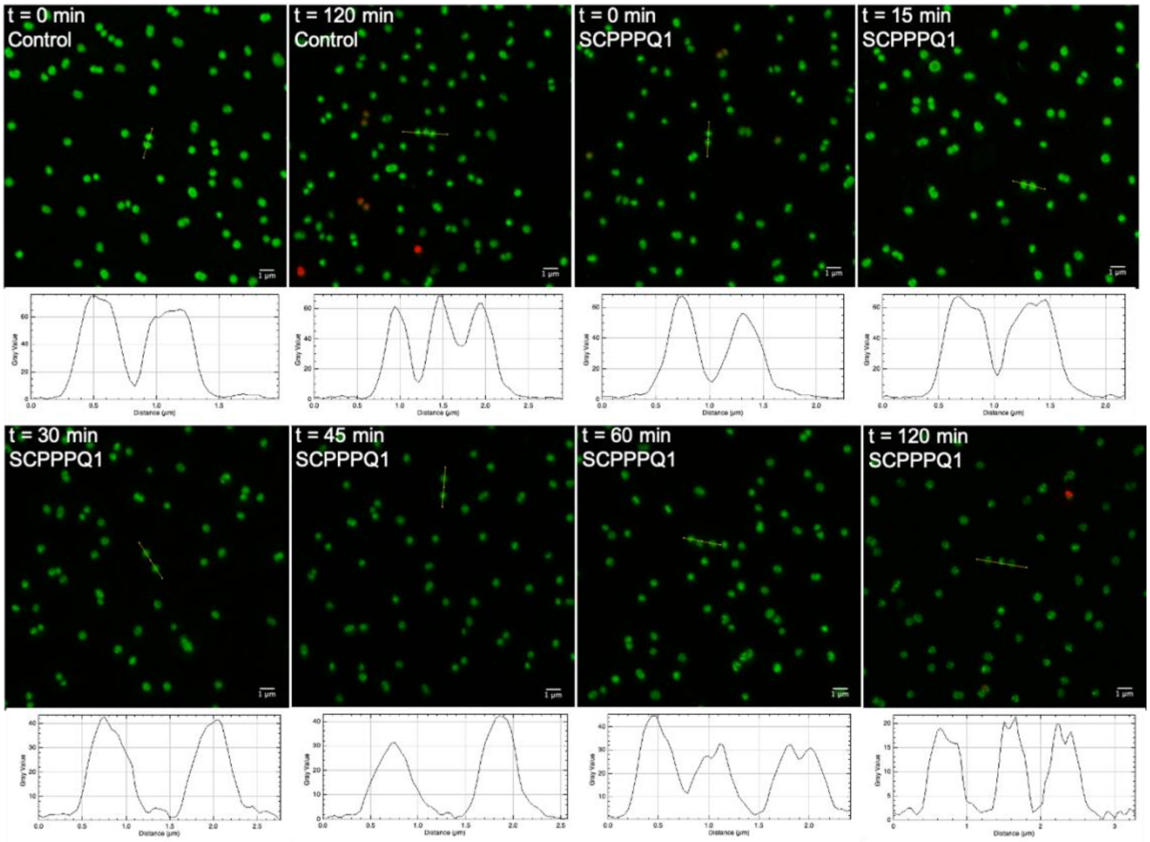


**Figure S3.** Fluorescence and image analysis of live-and-dead super-resolution images of *P. gingivalis* after incubation with the buffer only (control) or SCPPPQ1. Under each image are representatives plot profile intensity graph (gray values) of the line drawn across bacteria in the image panel. Over time, the fluorescence of the bacteria incubated with SCPPPQ1 decreased significantly (gray values decrease from ~70 to ~30 A.U.) compared to the ones incubated with the buffer (gray values ~70 A.U. throughout the incubation period). These diagrams are representative of the measure of more than 50 bacteria per conditions. Green = Syto9 labelling; Red = Propidium iodide. *A.U. = Arbitral unit*.

**Movie S1.** "FIB and view" analysis of *P. gingivalis* incubated with SCPPPQ1. Three-dimensional reconstructions reveal important membrane recesses on multiple bacteria.

**Movie S2.** "FIB and view" analysis of *P. gingivalis* incubated with the buffer. Three-dimensional reconstructions reveal generally smooth surfaces on the bacteria (compare with Movie S1).
